# Recent Advances in Fluorescent Probes for Cancer Biomarker Detection

**DOI:** 10.3390/molecules29051168

**Published:** 2024-03-06

**Authors:** Mingce Tian, Riliga Wu, Caihong Xiang, Guangle Niu, Weijiang Guan

**Affiliations:** 1 State Key Laboratory of Chemical Resource Engineering, Beijing University of Chemical Technology, Beijing 100029, China; 2 Beijing Institute of Smart Energy, Beijing 102209, China; 3 School of Chemistry and Chemical Engineering, Beijing Institute of Technology, Beijing 100081, China; xch200011033683@163.com

**Keywords:** cancer biomarkers, fluorescent probe, detection, biosensing, imaging

## Abstract

Many important biological species have been identified as cancer biomarkers and are gradually becoming reliable targets for early diagnosis and late therapeutic evaluation of cancer. However, accurate quantitative detection of cancer biomarkers remains challenging due to the complexity of biological systems and the diversity of cancer development. Fluorescent probes have been extensively utilized for identifying biological substances due to their notable benefits of being non-invasive, quickly responsive, highly sensitive and selective, allowing real-time visualization, and easily modifiable. This review critiques fluorescent probes used for detecting and imaging cancer biomarkers over the last five years. Focuses are made on the design strategies of small-molecule and nano-sized fluorescent probes, the construction methods of fluorescence sensing and imaging platforms, and their further applications in detection of multiple biomarkers, including enzymes, reactive oxygen species, reactive sulfur species, and microenvironments. This review aims to guide the design and development of excellent cancer diagnostic fluorescent probes, and promote the broad application of fluorescence analysis in early cancer diagnosis.

## 1. Introduction

The high mortality rate of cancer is due to the lack of typical morphologic structures in the early stages of cancer development, which fails to detect and diagnose cancer in time, thus causing patients to miss the optimal treatment period [1,2,3]. In fact, in the early stages of cancer development, changes occur inside the cell that are different from normal cells, such as increased expression of certain sugars, proteins, and hormones, as well as changes in pH, reactive oxygen species (ROS), and viscosity [4,5,6]. These substances, produced by malignant tumor cells or other cells in the body in response to conditions such as cancer, are called cancer biomarkers [7,8,9]. Therefore, detecting accurately and quantitatively cancer biomarkers is of great value for the early and accurate diagnosis of cancer and for evaluating the effectiveness of treatment in later stages.

Due to the complexity of biological systems, the detection and imaging of cancer biomarkers require high resolution, fast response, and deep imaging to provide a comprehensive view of biological fine structures [10,11]. Moreover, the development of cancer is complex with various changes in multiple cancer biomarkers [12]. In order to accurately determine the types and stages of cancer and its treatment status, analytical tests such as qualitative and quantitative tests and the localization of multiple cancer biomarkers need to be performed simultaneously. Among the developed detection methods, fluorescent probes are widely used in live cell imaging, drug delivery, and fluorescence-guided surgery due to their inherent advantages of non-invasiveness, fast response, high sensitivity and selectivity, real-time imaging, and flexible modifiability [13,14,15]. Thus, fluorescent probes provide endless possibilities to detect cancer biomarkers.

Probes for cancer biomarker detection typically consist of receptor/response sites, fluorescent components, and a communication mechanism between them to produce a detectable signal upon interaction with the targets [16,17]. The design of the fluorescent probe can be flexibly adapted to the test object and application environment, while selecting the appropriate fluorescence generation mode needs careful consideration [18]. In addition, fluorescent probes can be modified and customized to meet the detection requirement of a wide range of cancer biomarkers [19,20]. To better understand the advantage of fluorescent probes in detecting/imaging cancer biomarkers, this review summarizes the design and optimization strategies of small-molecule and nano-sized fluorescent probes and their working mechanisms for detecting cancer biomarkers. The applications for cancer marker detections in vitro and in vivo with representative examples are also presented. To conclude, we discuss the currently existing challenges and problems and share our understanding of future development directions of fluorescent probes and their promising detections for specific cancer markers in clinical applications. Hopefully, this review can help researchers move the field of cancer-marker fluorescent probes forward from bench to bedside.

## 2. Organic Small-Molecule Fluorescent Probes

Organic small molecule fluorescent probes are widely used in biological imaging and are characterized by synthesizing specific probe structures using pure organic chemistry. The versatile modifiability of the probe structures facilitates the continuous updating and optimization of their properties [11]. The emission wavelength of the probes can be red-shifted to the near-infrared (NIR) region by increasing the conjugated structure of the fluorescent moiety or by linking specific moieties [21,22,23]. The exquisite modifiability makes organic small molecule fluorescent probes widely used for cancer biomarker detection. This section summarizes the fluorescent moieties such as boron dipyrromethene (BODIPY), rhodamine, and tetraphenylethylene (TPE) that are commonly used in cancer biomarker detection. Their distinct advantages in detection are discussed below.

### 2.1. BODIPY-Based Fluorescent Probes

BODIPY dyes, part of the organoboron group, possess numerous outstanding optical characteristics including intense fluorescence, elevated molar extinction coefficient, and high fluorescence quantum yield [24,25]. BODIPY has a planar and stable core structure. With appropriate chemical modifications, BODIPY dyes can be spectrally tuned to cover from the green to NIR regions [26,27]. In addition, their remarkable biocompatibility makes them useful for diverse biological applications, including ion recognition, biomarker detection, and phototherapy [28,29]. 

**Figure 1:**
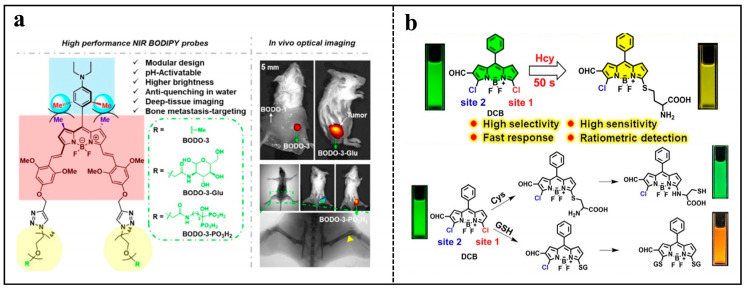
(**a**) Illustration showing the modular structure of pH-responsive NIR BODIPYs. Reproduced with permission from ref. [30]. Copyright 2021 American Chemical Society. (**b**) Proposed mechanisms of DCB reacting with Hcy, Cys, and GSH, respectively. Reproduced with permission from ref. [31]. Copyright 2022 American Chemical Society.

Various pH-responsive NIR BODIPY sensors were created with distinct axial groups to detect deep bone metastases of early breast cancer using non-invasive fluorescence in vivo (Figure 1a) [30]. These BODIPY probes consisted of an aniline substituent at the *meso* position and two PEG tails with different functions, serving as pH-sensitive unit and water-soluble group, respectively. These probes were capable of producing NIR fluorescence in a tumor acidic pH environment. Interestingly, the NIR BODO-3 could penetrate chicken breast tissue up to 8 mm. Further covalent functionalization with bisphosphonates enables BODO-3-PO_3_H_2_ to selectively target and visualize bone metastases of deep breast cancer earlier than X-rays in living mice.

BODIPY-based fluorescent probes can also distinguish different cancer biomarkers. A method based on using the bi-ligand strategy, a BODIPY probe named DCB was developed to selectively discriminate homocysteine (Hcy), cysteine (Cys), and glutathione (GSH) within 50 s with a low limit of detection (LOD) (Figure 1b). DCB showed a sufficient linear range of detection by means of different thermodynamics and reaction kinetics, enabling accurate real-time detection of Hcy in living cells. In addition, a positive correlation between aggregated amyloid-*β* (A*β*) peptides and changes in Hcy levels in neurons was confirmed by neuronal experiments [31].

### 2.2. Rhodamine-Based Fluorescent Probes

Rhodamine fluorophores show great potential for use in fluorescent probes, particularly in NIR rhodamine fluorescent probes, due to their outstanding photophysical characteristics, including strong absorption coefficients, exceptional fluorescence quantum yields, and robust structural stability [32,33]. The design of NIR rhodamine fluorescent probes involves two main strategies: replacing the oxygen bridges in the rhodamine backbone with other heteroatoms or a conjugated system of extended fluorophore molecules [34,35].

**Figure 2:**
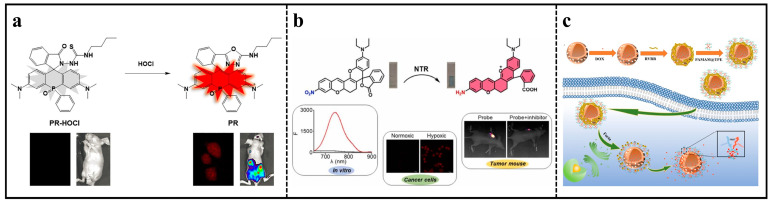
(**a**) Activate fluorescence in PR–HOCl by reacting with HOCl. Reproduced with permission from ref. [34]. Copyright 2020 Elsevier. (**b**) Sensing mechanism of near-infrared probe (ZY-2) for detecting NTR. Reproduced with permission from ref. [36]. Copyright 2022 Elsevier. (**c**) Schematic illustrations showing the process and mechanism of HMSN/DOX/RVRR/PAMAM/TPE nanoprobes. Reproduced with permission from ref. [43]. Copyright 2023 American Chemical Society.

A novel NIR fluorescent probe, PR-HOCl, with a maximum fluorescence emission wavelength of 730 nm, was developed by replacing the bridging oxygen in rhodamine with phosphorus (Figure 2a). The PR-HOCl exhibited rapid reactivity (20 s) and strong specificity towards hypochlorous acid (HOCl), with the LOD of 10 nM for the sensor. Furthermore, PR-HOCl was effectively utilized for identifying exogenous and endogenous hypochlorite ions in both the RAW264.7 cell line and inflammatory mice [34].

NIR nitroreductase (NTR)-sensitive fluorescent rhodamine probes (ZY-2) were synthesized by extending the *π*-conjugation system (Figure 2b). Without NTR, the spirocyclic ring-closed probe barely fluoresced. However, after reaction with NTR, the nitro group was specifically reduced to an amino group, rendering the spirocyclic ring opening and boosting NIR fluorescence (up to 32-fold). The intramolecular rotation of ZY-2 was restricted due to the formation of covalent bonds, which significantly enhanced the sensitivity and fluorescence quantum yield of the probe when reacted with NTR. In addition, the biocompatible ZY-2 could target mitochondria to evaluate the level of hypoxia in HeLa and HepG2 cells [36].

### 2.3. TPE-Based Fluorescent Probes 

Aggregation-induced emission (AIE) discovered by Tang et al. has become a widely used fluorescence technique [37,38]. AIE luminogens (AIEgens) are usually non-emissive in the molecular state, but show enhanced emission in the aggregated state due to the restriction of intramolecular motions [39,40]. Furthermore, AIEgens can be used at high concentrations without being affected by the aggregation-caused quenching phenomenon seen in traditional organic fluorescent dyes. Due to the unique advantages, AIE-based fluorescence imaging has been widely used for cancer diagnosis. Among the developed AIE molecules, the tetraphenylethylene (TPE) has received most extensive attention due to its superb fluorescence properties and facile modification [41,42].

In order to detect and treat triple-negative breast cancer early, a fluorescence resonance energy transfer (FRET)-based nanosized fluorescent probe was designed that can be catalytically initiated by a specific cleavage enzyme (Figure 2c). The specific scheme was as follows: hollow mesoporous silica nanoparticles (HMSN) were used as a carrier for loading doxorubicin (DOX), and the HMSN nanopore was encapsulated with an Arg-Val-Arg-Arg (RVRR) peptide, which responded specifically to Flynn. Furthermore, polyamylamine/phenylethane (PAMAM/TPE) was strongly attached to the external layer of HMSN, creating a FRET probe inside the cell. Upon entry into the tumor cell, the highly expressed Flynn enzyme cut the particular peptide sequence, leading to the release of DOX from the nanopore. Due to the tight binding of PAMAM/TPE to the outer surface of HMSN, an effective connection of DOX was achieved, which formed a certain adhesive force and hindered the rapid diffusion of DOX. The close proximity of molecules in TPE/DOX leads to a heightened FRET response, allowing for precise measurement of intracellular Flynn and indicating the release of DOX, ultimately facilitating drug delivery and tumor treatment [43]. 

## 3. Nano-Fluorescent Probes

Since the 21st century, nanotechnology has been developed rapidly, and a wide range of nanomaterials have been developed and applied. Nanomaterials possess distinct physicochemical characteristics that make them well-suited for biological monitoring and bioimaging [11,44]. Compared with small molecule fluorescent probes, nanomaterials have a large specific surface area with a variety of loaded signaling sensors, thereby reducing the LOD of the sensors. Second, nanomaterials can be chemically modified easily to enable multi-channel simultaneous detection of a single analyte or simultaneous detection of multiple analytes [45,46,47]. In this section, we present recent advances and applications of fluorescent nano probes such as metal nanoclusters (MNCs), fluorescent nanoparticles (FNPs), and quantum dots (QDs), for cancer biomarkers.

### 3.1. Fluorescent MNCs Nanoprobes 

MNCs are composed of 2–100 metal atoms, typically between 1–10 nm in size, and are sub-nanomaterials between atoms and nanoparticles [48]. They exhibit size-dependent fluorescence properties due to quantum confinement effects. MNCs have been widely used to detect biomolecules such as metal ions, reactive ROS, and DNA, as well as to determine extracellular pH [49,50].

**Figure 3:**
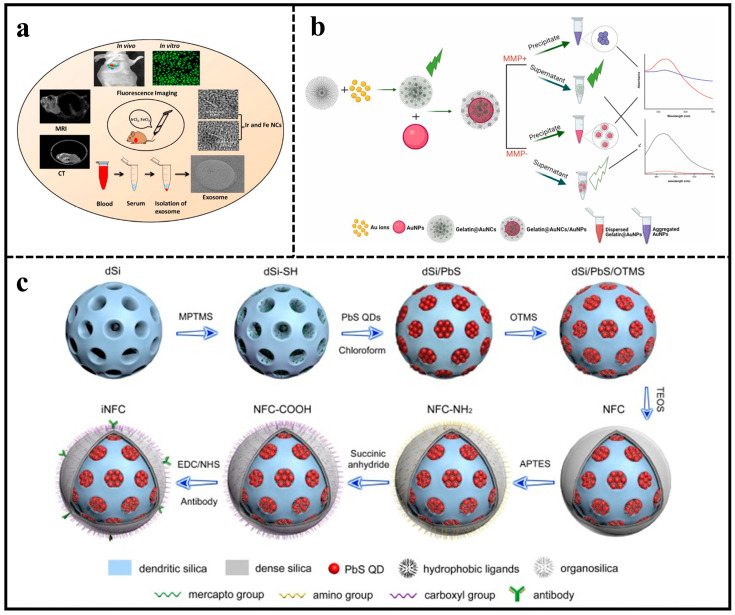
(**a**) Schematic illustration of the application of Ir and Fe NCs. Reproduced with permission from ref. [51]. Copyright 2018 American Chemical Society. (**b**) Proposed mechanism for detecting MMP-9 in dual mode using a nanocomposite platform of AuNPs@gelatin/AuNCs. Reproduced with permission from ref. [52]. Copyright 2023 Elsevier. (**c**) Schematic illustration of the synthetic procedure of NIR fluorescent colloid based nanoprobe. Reproduced with permission from ref. [62]. Copyright 2022 Elsevier.

Recently, MNCs have been developed for multimodal bioimaging to visualize the abnormal state of relevant disease target sites. The biosynthesis of iridium and iron oxide MNCs by facile in situ biosynthesis in cancer cells or tumor tissues was reported (Figure 3a). These MNCs were used as in vivo multimodal bioimaging probes to improve tumor-specific imaging. Observations showed that the highly luminescent and magnetic MNCs were biocompatible and tumor-targeting. In addition, biosynthesized MNCs and exosomes were isolated, which were shown to serve as biomarkers for cancer diagnosis [51].

Combining MNCs and NPs to develop fluorescent probes to detect cancer biomarkers is also a sensitive approach. For the highly sensitive detection of matrix metalloproteinases (MMPs), protein hydrolases are highly correlated with the development of malignant tumors. A gelatinase activity-based assay for MMP-9 was proposed (Figure 3b). The green-emitting gold nanoclusters (AuNCs) were synthesized using gelatin substrates and the AuNPs@gelatin/AuNCs nanocomposite structures were prepared by using gelatin/AuNCs-coated gold nanoparticles (AuNPs). The constructed nanocomposites exhibited the occurrence of internal FRET and fluorescence bursting of AuNCs due to the proximity of AuNCs/AuNPs structures. Matrix metalloproteinases caused alterations in the surface plasmon resonance of gold nanoparticles, leading to increased fluorescence from AuNCs by inhibiting the internal FRET process during hydrolysis of AuNPs@gelatin/AuNCs in saline buffer. The fluorescent probe was used for the MMP-9 enzyme with a naked-eye semiquantitative LOD of 2 ng/mL and a fluorescence-accurate detection LOD of 0.25 ng/mL [52]. 

### 3.2. FNPs Probes

There are two main types of FNPs: fluorescent organic nanoparticles (FONs) made up of organic polymers enclosing hydrophobic fluorophores through self-assembly, and inorganic FNPs doped with a monovalent anion or cation along with rare earth ions [53,54].

AuNPs with activatable fluorescent sensors are often used to construct novel biosensing systems. These sensors usually contain a fluorophore as a donor and a quench group as an acceptor. When the donor and acceptor are close to each other, the fluorescence can be quenched by energy transfer. This is consistent with the conditions of FRET, but nanosurface energy transfer (NSET) is not subject to the distance-dependent energy transfer limitations of FRET, making it more suitable for nanosensing systems. Hence, the sensor is designed by attaching different fluorophores to the AuNP surface in order to suppress the fluorescence of the fluorophore. Upon contact with the specific substance being analyzed, the sensor becomes active, causing the fluorescent molecule to detach from the surface of the tiny particle [11,55,56,57]. 

### 3.3. QDs Nanoprobes

QDs are nanoparticles that have a dimension of zero, made of semiconductor materials that are smaller than or similar to the exciton Bohr radius, showing high optical stability and size-dependent fluorescence properties. In 1998, Nie and Alivisatos first reported fluorescent QD probes by combining with biomolecules for cellular imaging [58]. Novel QDs with excellent optical properties and biocompatibility offer more options and broader application prospects for biomedical imaging. For example, NIR-emitting QDs can reduce background noise and penetrate deep into tissues, suitable for in vivo imaging and deep tissue imaging; cadmium-free QDs are conducive to reducing the impact on the environment and cytotoxicity; and carbon QDs have excellent optical properties and good biocompatibility, which are expected to become a new type of biomedical imaging probe [59,60].

Purine/pyrimidine-free endonuclease 1 (APE1), a crucial DNA repair enzyme that lacks purine and pyrimidine, is upregulated in the majority of cancer types and serves as a biomarker for cancer prediction and diagnosis. To monitor its enzymatic activity, a functional nanocomposite was meticulously constructed using a single molecule of DNA and fine-sized graphene QDs. Utilizing the nanocomposite as a diagnostic probe, APE1 could activate its fluorescence in a small number of cells. Most importantly, the construction ensured that these graphene QD-based nanocomposites could detect the same type of cancer biomarker APE1 under different cellular conditions. Moreover, this nanocomposite was applied to a wide range of cancer cells with high sensitivity and specificity [61].

Early cancer screening requires the simultaneous identification of various tumor biomarkers in serum. In order to detect multiple cancer markers at the same time, PbS QD emitting in the NIR-II range were created to minimize interference from the sample matrix (Figure 3c). Lateral-flow immunoassay (LFIA) test strips successfully detected the presence of carcinoembryonic antigen (CEA), keratin 19 fragment (Cyfra21-1), and neuron-specific enolase (NSE) simultaneously. The LFIA targeting multiple analyses can be finished in under 12 min, providing accurate quantitative results using a portable NIR-II strip scanner. Analysis data indicated that levels of CEA, Cyfra21-1, and NSE ranged from 0.11 to 100 ng/mg, 0.18 to 100 ng/mL, and 100 ng/mL, respectively, with an LOD of 0.11, 0.18, and 0.28 ng/mL. Furthermore, when testing actual serum samples from lung cancer patients and healthy individuals, the sensitivity of the combined assay using three biomarkers rose to 92.7%, surpassing the sensitivity of any single biomarker assay [62].

## 4. The Detection Application of Fluorescent Probes for Different Cancer Biomarkers

### 4.1. Fluorescent Probes for Different Enzymes

Enzymes, primarily as specific catalysts for myriad biochemical reactions to maintain homeostasis, play key roles in living systems [63,64]. Under normal conditions, enzyme activity and expression are stable in the body. However, if there are pathological alterations in organs or tissues, certain enzymes may exhibit abnormal elevation in activity. Recent research demonstrated a frequent correlation between cancer and atypical enzyme activity levels [65,66,67]. Therefore, it is crucial for researchers to create fluorescent probes at a small scale that can observe enzyme functions in real-time and without causing harm, in order to enhance their comprehension of the biological and pathological functions of enzymes. In this section, recent advances in the use of fluorescent probes for detecting or imaging the function of enzyme activity in living systems are discussed.

#### 4.1.1. Fluorescent Probes for Glycosidases

Glycosylation involves attaching polysaccharides to biomolecules (e.g., proteins and lipids) using glycosyltransferases, while deglycosylation involves removing sugars from biomolecules using glycosidases. The advancement of several diseases, such as cancer, neurodegenerative diseases, lung diseases, blood disorders, and genetic disorders, has been linked to protein glycosylation [17,68,69,70]. Glycosylation and deglycosylation of biomolecules are regulated by glycosyltransferases and glycosidases, respectively.

**Figure 4:**
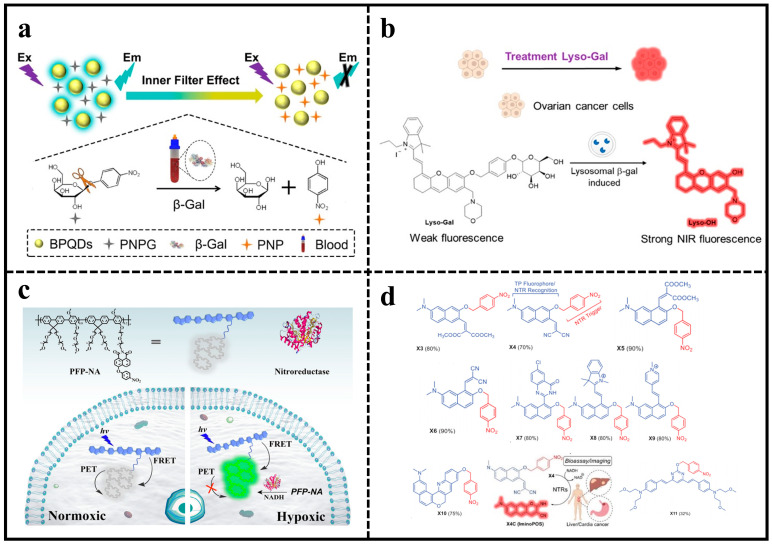
(**a**) Illustration showing the design of a sensor using IFE for detecting *β*-Gal with BPQDs. Reproduced with permission from ref. [72]. Copyright 2019 Elsevier. (**b**) Schematic illustration of Lyso-Gal sensor for detection of *β*-Gal. Reproduced with permission from ref. [73]. Copyright 2020 American Chemical Society. (**c**) The detection mechanism of PFP-NA towards NTR. Reproduced with permission from ref. [76]. Copyright 2020 Elsevier. (**d**) Synthetic pathways for NTR-detecting TPFPs (X1-X11) and the operational principle of X4 in relation to NTR. Reproduced with permission from ref. [77]. Copyright 2022 John Wiley and Sons.

*β*-Galactosidase (*β*-Gal) is a widely known glycosidase that selectively removes galactose units from glycosylation complexes, plays a vital role in the regulation of various physiological processes, and is considered one of the most important biomarkers of primary ovarian cancer and cellular senescence [71]. For the selective detection of *β*-Gal, a simple and direct platform for the single-step detection of ovarian cancer-associated glycosidase activity was developed based on the internal filtration effect (IFE) between glycosidase catalytic products and black phosphorus QDs (BPQDs) (Figure 4a). Under the influence of *β*-Gal, p-nitrophenyl-*β*-D-galactopyranoside (PNPG) underwent a transformation into p-nitrophenol (PNP) and *β*-D-galactopyranoside. The fluorescence of the BPQDs was quenched as a result of the significant overlap between the absorption of the catalytic product PNP and the emission spectra of the BPQDs. The technique exhibits a low LOD (0.76 U/L), significantly lower than the majority of detection systems by 1–2 orders of magnitude. The feasibility of the sensing platform for screening potential inhibitors was further evaluated by selecting D-galacturonic acid as an inhibitor of *β*-Gal, showing much potential in the evaluation of inhibitors of *β*-Gal [72].

For more precise detection of *β*-Gal, Lyso-Gal, an NIR fluorescent probe with lysosome-targeting capabilities, was developed for detecting and imaging *β*-Gal in lysosomes within ovarian cancer cells (SKOV-3 cells) (Figure 4b). Lyso-Gal showed low fluorescence, but after being incubated with *β*-Gal, it displayed a strong NIR fluorescence at 725 nm, demonstrating high selectivity and sensitivity. Lyso-Gal displayed superior fluorescence compared to Hx-Gal, enabling the visualization of endogenous *β*-Gal in lysosomes of SKOV-3 cells [73].

#### 4.1.2. Fluorescent Probes for Nitroreductases

Nitroreductase (NTR) is a common biomarker of hypoxic enzymes and is widely used in assessing the tumor microenvironment [74,75]. A new ratiometric fluorescent biosensor (PFP-NA) utilizing a soluble conjugated polymer was created to detect and diagnose tumor hypoxia effectively for NTR (Figure 4c). The sensor enabled FRET and photoinduced electron transfer (PET) to occur simultaneously by covalently bonding p-nitrophenyl-modified 1,8-naphthimide (NA) to the side chain of poly(fluorene-co-phenylene) (PFP). Due to PET quenching, the fluorescence intensity of NA was reduced, resulting in a low emission ratio of NA compared to the PFP backbone. In the presence of NTR, the nitro group was converted to an amino group, leading to PET inhibition and a significant increase in emission ratio (>54), with a calculated LOD as low as 19.7 ng/mL [76]. Furthermore, the researchers developed a set of two-photon fluorescent probes that can detect NTR activity in different biological samples with high sensitivity using computational modeling (Figure 4d). By optimizing the distance between the reaction sites of NTRs from different sources, X4 was found to show the best performance in terms of both sensitivity and selectivity. The excellent two-photon excitation fluorescence properties of X4 enabled it for direct monitoring and imaging of endogenous NTR activity in live mammalian cells, growing zebrafish, and hormonal mice with low background autofluorescence interference [77].

### 4.2. Fluorescent Probes for ROS/RSS

Reactive oxygen species (ROS, e.g., hydrogen peroxide (H_2_O_2_), HOCl), and reactive sulfur species (RSS, e.g., GSH, Cys) play a crucial role in the body. These substances are usually highly reactive and play a key role in maintaining homeostasis in the intracellular environment. Under normal physiological conditions, these reactive substances are in dynamic equilibrium. However, in cases of pathological alterations or heightened oxidative stress, there is an abnormal rise in the levels of these compounds. Furthermore, the excessive production of these potent compounds is frequently linked to a range of health conditions, including inflammation, neurodegenerative disorders, and cancer [78,79,80,81]. To better understand and monitor the changes of these active substances in biological systems, researchers have designed and synthesized diverse fluorescent probes.

#### 4.2.1. Fluorescent Probes for H_2_O_2_

H_2_O_2_ is an important ROS and a key marker of oxidative stress in various diseases. Visualizing H_2_O_2_ in vivo is crucial in disease diagnosis, but H_2_O_2_ monitoring remains challenging due to the autofluorescence of biological samples.

**Figure 5:**
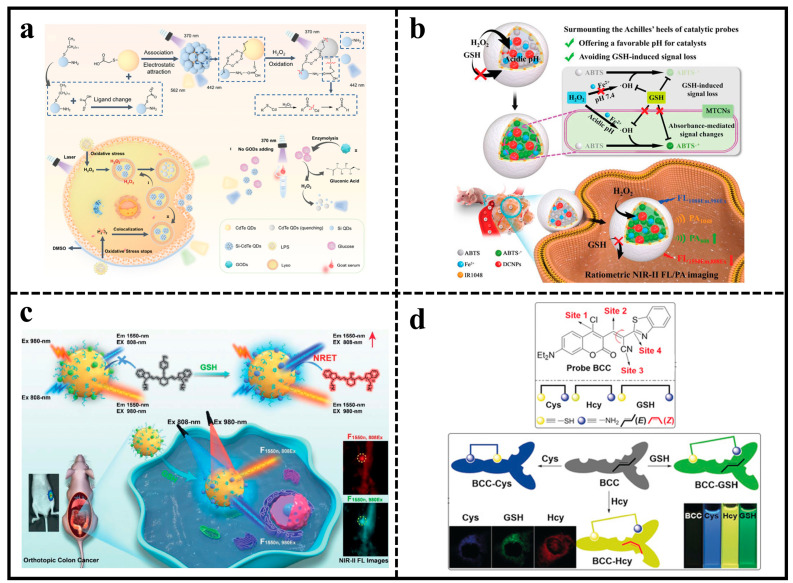
(**a**) A diagram illustrating the creation of Si-CdTe QDs probe for detecting H_2_O_2_ in cells and glucose in goat serum, respectively. Reproduced with permission from ref. [82]. Copyright 2022 John Wiley and Sons. (**b**) Illustration of MTCNs for ratiometric NIR-II fluorescence/photoacoustic (FL/PA) imaging of H_2_O_2_ in tumor and lymphatic metastasis. Reproduced with permission from ref. [83]. Copyright 2022 John Wiley and Sons. (**c**) NPh-conjugated lanthanide-based DCNP schematic for ratiometric detection of GSH using NIR-II FL nanoprobe and NRET effect. Reproduced with permission from ref. [87]. Copyright 2021 John Wiley and Sons. (**d**) Detection of Cys, Hcy, and GSH at the same time using the four binding sites of probe BCC. Reproduced with permission from ref. [91]. Copyright 2018 John Wiley and Sons.

To monitor H_2_O_2_ in vivo in real time, a hydrophilic ratiometric fluorescent probe Si-CdTe QDs with bimodal emission was created by combining CdTe QDs with Si QDs (Figure 5a). The LOD of the probe was as low as 79 and 140 nM for H_2_O_2_ and glucose, respectively. The cell viability remained 80% when HeLa cells were co-cultured with Si-CdTe QDs at a concentration of 500 µg/mL for 24 h. The visualization of intracellular H_2_O_2_ distribution in lysosomes with low background interference was achieved by using an excitation light at 488 nm and a fluorescence channel at 514 nm. The Si-CdTe QDs probe provided a new and convenient way to monitor the changes of intracellular H_2_O_2_ concentration [82].

While probes utilizing catalytic reactions show promise for detecting H_2_O_2_, their in vivo application is difficult due to the need for catalysts to operate at a specific pH and the potential inhibition of signaling by intracellular GSH through the depletion of hydroxyl radicals and oxidation of substrates. Fe^2+^, citric acid (CA), 2,2′-azino-bis(3-ethylbenzothiazole-6-sulfonic acid) ammonium salt (ABTS), and down-converted nanoparticles were incorporated into both the liposome interior and the lipid membranes to create a customized catalytic nanoprobe (MTCN) in specific conditions (Figure 5b). The selective permeability of the MTCN to the liposome membranes could provide a favorable pH environment. It not only enhanced Fe^2+^ activity, but also avoided signal loss due to GSH triggering by inhibiting GSH entry into the lumen. Furthermore, upon exposure to H_2_O_2_, ABTS was converted to ABTS^+^ with 808 nm absorption caused a noticeable increment in PA_808_/PA_1048_ accompanied by an apparent decrement in FL_1550Em,808Ex_/FL_1080Em,980Ex_. This enabled the bimodal ratiometric fluorescent probe to detect H_2_O_2_ in tumors [83].

#### 4.2.2. Fluorescent Probes for GSH

Recent research indicates that GSH is associated with various illnesses, such as cancer and Alzheimer’s disease. Therefore, the monitoring of GSH is crucial for accurate feedback on intracellular redox status and real-time visualization of physiological and pathological conditions in vivo [84,85,86]. However, traditional NIR fluorescence imaging (NIR-I, 650–900 nm) faces challenges in accurately visualizing in vivo because of intense background fluorescence and photon scattering. To avoid the background fluorescence and photon scattering, an NIR-IIb (1500–1700 nm) nanoprobe consisting of lanthanide down-conversion nanoparticles conjugated with 4-nitrophenol-Cy7 (NPh) was developed for ratiometric imaging of GSH in vivo (Figure 5c). After reaction with GSH, the probe emitted strong fluorescence emission at 808 nm, which enhanced the fluorescence signal of DCNPs at 1550 nm through the non-radiant energy transfer (NRET) effect. The emission ratio (Em_1550_, Ex_980_/Em_1550_, Ex_808_) correlated linearly with GSH concentration in the range of 0–24 mM, with a LOD of 0.3 mM. The NIR-IIb nanoprobe accurately detected and imaged GSH in subcutaneous tumors and in situ colon tumors in vivo with high resolution [87].

Considering the distinct physiological roles of biothiols in organisms and the tight relationship between many diseases and changes in their concentrations (Cys: 30–200 μM; GSH: 1–10 mM), the development of effective methods for real-time monitoring of intracellular Cys, Hcy, and GSH concentrations is of great value [88,89,90]. A novel chlorocoumarin–benzothiazole fluorescent probe with four binding sites was designed and synthesized using coumarin as a fluorophore (Figure 5d). The probe can simultaneously detect Cys, Hcy, and GSH in three different fluorescence emission channels. Depending on the different reaction mechanisms, the fluorescence increments of Cys, Hcy, and GSH were 108-fold, 128-fold, and 30-fold at 457, 559, and 529 nm, respectively. In addition, multicolor imaging studies confirmed that the probe can be used for simultaneous monitoring of endogenous Cys, GSH, as well as exogenous Cys, Hcy, and GSH in cells. The exploration provided a feasible way to further explore the cellular dynamics of thiols in organisms and their functions in biological systems, contributing to the study of thiols in biomedicine and diagnostics [91].

### 4.3. Fluorescent Probes for Cancer Microenvironmental Factors

A dynamically stable environment inside the cell is essential to maintain normal physiological functions. Every organelle needs an appropriate surrounding environment, including pH, viscosity, and polarity to operate effectively. However, the progression of numerous types of cancer frequently involves alterations in the surrounding environment, such as rising viscosity and declining pH levels. Therefore, abnormal changes in the tumor microenvironment can be important indicators for cancer diagnosis [92,93,94].

#### 4.3.1. Fluorescent Probes for pH

Among substances of clinical concern, pH is critical for regulating acidity and alkalinity. Rapid fluctuations in pH can exert significant control over cellular, organ, and organismal functions. For example, changes in pH can predict cancer growth and heart disease. Cell death and excessive inflammation can lead to acidification in unhealthy tissues. Tissue acidosis is also a hallmark of inflammatory disease [95,96,97].

**Figure 6:**
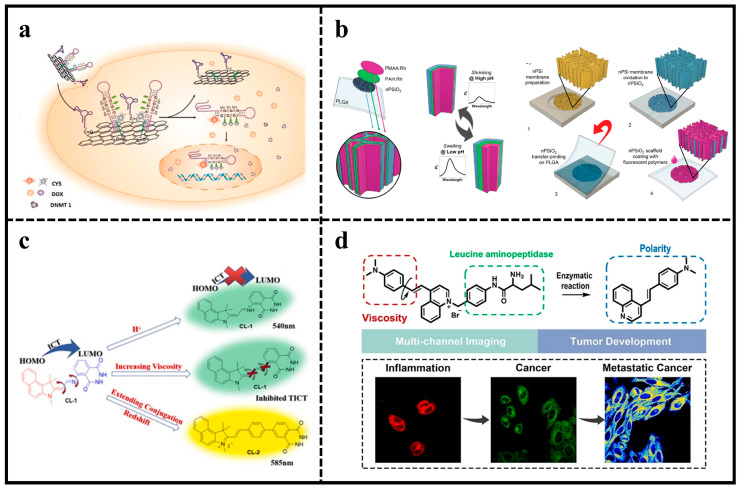
(**a**) Demonstration of pH-sensitive cancer-specific fluorescent imaging and dual treatment using DNA-linked graphene oxide. Reproduced with permission from ref. [98]. Copyright 2022 Elsevier. (**b**) Preparation of the bioresorbable fluorescence pH sensor. Reproduced with permission from ref. [99]. Copyright 2022 John Wiley and Sons. (**c**) Development of fluorescent probes that respond to both pH and viscosity through design strategy. Reproduced with permission from ref. [103]. Copyright 2023 Elsevier. (**d**) Illustration of the fluorescent probe VLAP for simultaneously monitoring the mitochondrial viscosity, polarity, and LAP. Reproduced with permission from ref. [104]. Copyright 2022 Elsevier.

A novel cancer therapeutic nano-assembly with high biocompatibility, stability, and low toxicity was developed, which can be rapidly activated by the tumor acidic microenvironment for selective fluorescence imaging, chemotherapy, and chemo-enzymatic therapy (Figure 6a). The nanoprobes were synthesized by hybridization of hairpin DNA labeled with a fluorophore containing 5-aza-dC at the hemi-methylated CpG site and pH-sensitive DNA sequences covalently coupled with polyethylene glycolated graphene oxide (GO). Nucleic acid aptamers were also covalently coupled to the polyethylene glycolate GO and could target the tumor site. The weakly acidic environment of the tumor may induce the liberation of medications carried by nanoprobes, such as modified DNA and DOXs, effectively initiating glowing signals and specifically eliminating cancerous cells [98].

Recently, a bioresorbable nanostructured sensor was developed for pH sensing. The sensor was made up of a thin, porous silicon membrane layered with a thin multilayer membrane and two polyelectrolytes containing pH-insensitive fluorophores (Figure 6b). When the polymer multilayer membrane expanded/contracted, the fluorescence of the sensor changed linearly with a pH range of 4–7.5, enabling real-time pH measurements for 100 h with high stability, reproducibility, and accuracy. An in vivo exploration of the sensor implanted subcutaneously on the back of mice confirmed the ability of the sensor to monitor local pH through the skin in real time. In addition, the pH sensor completely degraded within one week after implantation in animals, and its good biocompatibility was confirmed by histological and fluorescence analyses after 2 months [99].

#### 4.3.2. Fluorescent Probes for Viscosity

Intracellular viscosity is another critical parameter in the cellular microenvironment and has a significant role in various intracellular interactions, such as signal transduction and diffusion of various substances. Unusual thickness often results in metabolic issues and illnesses, such as cancer, diabetes, and atherosclerosis [100,101,102]. 

Molecular rotors in fluorophores enable the measurement and continuous tracking of changes in micro-viscosity in living cells. The non-radiative decay of fluorescent rotors in the excited state is affected by viscosity changes. In high-viscosity environments, intramolecular rotation is hindered, resulting in high quantum yields and long fluorescence lifetimes. Thus, by targeting intramolecular charge transfer (ICT) and twisting intramolecular charge transfer (TICT), two dual-mode fluorescent sensors (CL-1 and CL-2) were created to detect changes in pH and viscosity. The maximum fluorescence wavelengths for CL-1 and CL-2 in acidic and high-viscosity conditions were 540 nm and 585 nm, respectively (Figure 6c). The fluorescence intensities of probes were strongly correlated with pH and viscosity. Density functional theory calculations showed that the fluorescent probes achieved fluorescence enhancement at low pH and high viscosity due to ICT and TICT. The imaging ability of probes in living cells and tumors was also demonstrated by bioimaging experiments [103].

Simultaneous multicolor imaging of multiple analyses is essential for biological research. Despite the development of numerous fluorescent probes for identifying individual components, simultaneous imaging for multiple components with one small-molecule probe remains challenging, due to complicated molecular design and potential optical crosstalk. Toward this end, a single fluorescent probe VLAP with a large Stokes shift was reported for simultaneously monitoring the mitochondrial viscosity, polarity, and leucine aminopeptidase (LAP) in cells under different pathophysiological conditions through different fluorescence channels using LAP as a “switch” (Figure 6d). The probe can detect changes in viscosity (50-fold fluorescence enhancement), LAP (15-fold fluorescence enhancement), and polarity (20-fold ratio change) with high sensitivity due to its optical properties, all without interference. Experimental studies confirmed the ability to detect up-regulation of viscosity in inflammatory cells, the kinetics of LAP in hepatocellular carcinoma cells, and the lower polarity of mitochondria [104].

## 5. Conclusions

Detecting cancer biomarkers has emerged as an essential approach to early diagnosis and evaluation of cancer. Fluorescent probes have emerged as a powerful method for identifying and observing biological entities in recent times, due to their exceptional benefits. High-performing fluorescent markers can fulfill the needs for non-invasive, real-time, accurate, and targeted imaging of intricate internal structures and biochemical activities, enabling quick and sensitive identification of cancer biomarkers both qualitatively and quantitatively. In this review, we summarized recent advances in organic small-molecule and nano-sized fluorescent probes and their fluorescence sensing imaging platforms. In particular, we explored biomarkers suitable for detecting cancer through various fluorescent probes, providing illustrative instances. 

Despite these considerable progresses achieved, several issues still limit their use in early cancer diagnosis: (1) To transition the tumor diagnosis process from laboratory settings to live subjects, the challenge of inadequate tissue penetration due to short wavelengths must be addressed. Developing fluorescent probes with two-photon absorption or with NIR-II emissive properties is required. However, the balance between emission brightness and fluorescence wavelength needs careful consideration. (2) The specificity of probes toward cancer regions is highly demanding for accurate diagnosis, which requires the introduction of an alternative targeting group on the probe skeletons by utilizing specific receptors overexpressed in cancer cells. (3) The short retention and rapid metabolism of the probes after activation fail for long-term imaging and monitoring of the development of cancers in the body. To overcome this problem, some immobilization strategies should be considered to maintain tumor imaging over a long period. In addition, some important parameters, such as stability, biotoxicity, and metabolic kinetics, must be considered when designing probes to diagnose cancer cells/tissues so that they can be practically used in clinical practice in the future. Overall, the development of fluorescent probes is still in the basic research stage, and there is still a lot of work to be carried out to apply these discoveries in the clinic. It is believed that with the development of multifunctional fluorescent probes, these critical issues will be gradually resolved for more accurate cancer diagnosis, making outstanding contributions to the cause of precision medicine.

## Data Availability

Not applicable.
